# Preliminary Study on the Evaluation Value of Extracellular Volume Fraction in the Pathological Grading of Lung Invasive Adenocarcinoma

**DOI:** 10.2174/0115734056392707250818063540

**Published:** 2025-08-26

**Authors:** Bin Nan, Yukun Pan, Yinghui Ge, Minghua Sun, Jin Cai, Xiaojing Kan

**Affiliations:** 1 Department of Radiology, Fuwai Central China Cardiovascular Hospital, Henan Provincial Key Laboratory of Cardiology Medical Imaging, Zhengzhou, Henan 450003, China

**Keywords:** Lung neoplasms, Adenocarcinoma, Tomography, X-ray computed, Dual-energy CT imaging, Differential diagnosis, ECV_A_

## Abstract

**Introduction:**

This study aims to evaluate the diagnostic value of extracellular volume fraction (ECV) and spectral CT parameters in assessing the pathological grading of lung invasive adenocarcinoma (IAC) presenting as solid or subsolid nodules.

**Methods:**

A retrospective collection of patients who were pathologically confirmed as IAC with solid or subsolid pulmonary nodules at our hospital from March 2023 to November 2024 was conducted. Relevant data were recorded, and the patients were divided into two groups: intermediate/high differentiation and low differentiation. The parameters including arterial phase iodine concentration (IC_A_), arterial phase normalized iodine concentration (NIC_A_), arterial phase normalized effective atomic number (nZeff_A_), arterial phase extracellular volume fraction (ECV_A_), venous phase iodine concentration (IC_V_), venous phase Normalized Iodine Concentration (NIC_V_), venous phase normalized effective atomic number (nZeff_V_), and venous phase extracellular volume fraction (ECV_V_) were compared between the two groups. Parameters with statistical significance were evaluated for their diagnostic performance using Receiver Operating Characteristic (ROC) curves.

**Results:**

A total of 61 patients were included, comprising 40 in the intermediate to high differentiation group and 21 in the low differentiation group. The intermediate/high differentiation group had higher values of ECV_A_, NIC_A_, ECV_V_, IC_V_, NIC_V_, and nZeff_V_ than the low differentiation group (*P* < 0.05). The AUC values for these parameters were 0.679, 0.620, 0.757, 0.688, 0.724 and 0.693 respectively. Among these, ECV_V_ had the largest AUC, with a sensitivity and specificity of 72.5% and 71.4%, respectively. Through binary logistic regression analysis, five features were identified: the maximum diameter of the lesion, bronchus encapsulated air sign, lobulation sign, spiculation sign, and pleural traction sign. The integration of these imaging features with ECV_V_ resulted in a model with enhanced diagnostic performance, characterized by an AUC of 0.886, a sensitivity of 85.7%, and a specificity of 80.0%.

**Discussion:**

ECV_V_ outperforms other spectral parameters in differentiating IAC grades, reflecting changes in the tumor microenvironment. Combining ECV_V_ with imaging features enhances diagnostic accuracy, though the study’s single-center design and small sample size limit generalizability.

**Conclusion:**

Extracellular volume fraction can provide more information for the pathological grading assessment of invasive adenocarcinoma of the lung. Compared to other spectral parameters, ECV_V_ exhibits the highest diagnostic performance, and its combination with conventional imaging features can further enhance diagnostic accuracy.

## 1 INTRODUCTION

Lung cancer remains one of the leading causes of cancer-related morbidity and mortality worldwide [1]. Among its various subtypes, invasive adenocarcinoma (IAC) represents the predominant pathological type of non-small cell lung cancer, with its incidence showing a continuous upward trend over the past decades [2]. The degree of differentiation in IAC is closely associated with its biological behavior, treatment response, and prognosis [3]. Studies have demonstrated that, compared to moderately and well-differentiated IACs, which typically exhibit slower growth patterns, poorly differentiated or undifferentiated tumors are characterized by more rapid progression, enhanced invasiveness, and a poorer response to conventional therapeutic approaches, including chemoradiotherapy [4]. Therefore, accurate assessment of IAC differentiation grade holds substantial clinical significance in tailoring individualized treatment strategies and predicting patient outcomes.

Currently, the evaluation of IAC differentiation primarily relies on histopathological examination, necessitating invasive procedures such as biopsy or surgery to obtain tumor tissue samples. However, this approach has notable limitations: firstly, the invasive nature of biopsies carries risks of complications, including hemorrhage and infection; secondly, sampling errors may occur in some biopsy procedures, potentially leading to discrepancies between the assessment results and the actual pathological grades [4]. Consequently, developing reliable non-invasive assessment methods holds paramount importance in improving patient care experiences and diagnostic accuracy [5]. In recent years, the rapid advancement of Dual-energy CT (DECT) technology has opened new avenues for the non-invasive evaluation of lung tumors [6]. The dual-layer detector spectral CT (DLCT) represents a significant advancement in DECT implementation, utilizing a single X-ray tube with two layers of detectors to simultaneously acquire low- and high-energy datasets [7, 8]. This technology not only provides morphological characteristics but also enables quantitative analysis through multiple parameters, including effective atomic number (Zeff), Iodine Concentration (IC), and extracellular volume fraction (ECV) [9].

ECV, as a quantitative parameter derived from equilibrium contrast-enhanced CT images and calculated with hematocrit, provides valuable information about changes in the cellular microenvironment [10]. Traditionally, ECV measurements have been primarily utilized in the assessment of cardiovascular diseases, organ fibrosis (such as pancreatic and hepatic fibrosis), and various abdominal cancers [11, 12]. Recent studies have demonstrated the potential value of ECV in differentiating lung cancer from benign lung lesions, as it effectively reflects tissue fibrosis characteristics [13]. In current clinical practice, chest contrast-enhanced CT protocols typically include arterial and venous phases after the administration of a contrast agent, making ECV measurement feasible in routine diagnostic imaging.

Although ECV has shown potential in distinguishing lung cancer from benign pulmonary lesions, its role in assessing the pathological differentiation of IAC has not been systematically investigated. Existing literature primarily focuses on the application of ECV in cardiovascular diseases and abdominal tumors. At the same time, studies on the differentiation grading of lung adenocarcinoma are extremely limited, with only preliminary explorations and a lack of systematic evaluation specifically targeting moderately to well-differentiated versus poorly differentiated IAC [13-15].

## 2 MATERIALS AND METHODS

### 2.1 Patients

This retrospective study included patients with surgically confirmed IAC treated at our institution between March 2023 and November 2024. Patient demographics (age, sex, and BMI) were recorded, along with hematocrit (Hct) values obtained within one week prior to the CT examination for subsequent calculations.

The inclusion criteria were: (1) surgically confirmed IAC; (2) no prior treatment before surgery; (3) at least one DECT examination performed within one month before surgery. Exclusion criteria comprised: (1) incomplete clinical or imaging data, including missing key clinical indicators such as age, sex, Body Mass Index (BMI), and preoperative hematocrit (Hct) values; (2) CT artifacts interfering with lesion observation and measurement; (3) nodules larger than 3 cm in diameter on thin-slice CT or non solid nodules; (4) any antitumor therapy prior to scanning.

### 2.2 Pathological Grading

IAC was classified into three prognostically relevant grades according to the 2015 World Health Organization (WHO) classification of lung adenocarcinoma: well-differentiated (Grade 1, predominantly lepidic pattern), moderately differentiated (Grade 2, predominantly acinar or papillary pattern), and poorly differentiated (Grade 3, predominantly solid or micropapillary pattern).

#### 2.2.1 CT Imaging Protocol

All examinations were performed using a dual-layer spectral detector CT scanner (Spectral CT 7500, Philips Healthcare, Netherlands) with the following parameters: tube voltage 120 kV; tube current modulated by automatic exposure control; detector collimation 128×0.625 mm; rotation time 0.5 seconds; pitch 1.0; reconstruction slice thickness 1 mm; and matrix 512×512. For contrast-enhanced scanning, non-ionic iodinated contrast medium (Ultravist 370 mg/ml, Bayer Schering Pharma) was administered intravenously through a power injector at a dose of 1 ml/kg body weight and a flow rate of 3.0 ml/s, followed by a 30 ml saline flush at the same rate. Using the bolus-tracking technique, arterial phase scanning was triggered 8 seconds after the attenuation threshold of 150 HU was reached in the descending aorta, with venous phase imaging performed 40 seconds after the arterial phase.

#### 2.2.2 Image Analysis

Spectral-Based Images (SBI) were transferred to the Philips IntelliSpace Portal workstation for quantitative analysis. Regions Of Interest (ROIs) were drawn on the maximum cross-sectional area of each lesion, encompassing at least two-thirds of the lesion area while carefully avoiding cavitation, calcification, and large vessels. For each lesion, measurements were obtained from three consecutive slices containing the maximum cross-section, and the mean values were recorded.

Quantitative parameters were measured and calculated for both arterial and venous phases, including Iodine Concentration (IC), Normalized Iodine Concentration (NIC), effective atomic number (Zeff), and ECV. The normalized effective atomic number (nZeff) was calculated as the ratio of lesion Zeff to aortic Zeff at the same level, while NIC was determined as the ratio of lesion IC to aortic IC at the corresponding level. The ECV was calculated using the following formula:

ECV (%) = (1 - Hct[%]) × (I_Clesion_/I_Caorta_) × 100%

All measurements and image analyses were performed independently by two radiologists with over five years of diagnostic experience. Inter-observer agreement was assessed, and mean values were used for statistical analysis when agreement was achieved.

### 2.3 Statistical Analysis

Statistical analyses were performed using SPSS version 27.0 and the R software. Continuous variables with normal distribution were expressed as mean ± standard deviation, while categorical variables were presented as percentages.

Inter-observer agreement for qualitative imaging features (lobulation, spiculation, pleural traction sign, bronchus encapsulated air sign) was evaluated using Cohen’s Kappa, and for quantitative DECT parameters (ECV_A_, ECV_V_, IC_A_, IC_V_, NIC_A_, NIC_V_, nZeff_A_, nZeff_V_) using the Intraclass Correlation Coefficient (ICC) based on a two-way random-effects model with absolute agreement. Kappa and ICC values greater than 0.75 indicated good reliability, with values between 0.61 and 0.80 classified as substantial and those between 0.81 and 1.00 as almost perfect.

An independent samples t-test was used for normally distributed continuous variables, while the Mann-Whitney U test was applied for non-normally distributed data. Categorical variables were compared using the chi-square test. Variables with *P* < 0.1 in the univariate logistic regression analysis were subsequently used to construct the imaging features model based on conventional imaging features.

Receiver Operating Characteristic (ROC) curves were generated to evaluate the diagnostic performance of statistically significant parameters, and Areas Under the Curve (AUC) were calculated. The parameter with the highest AUC was combined with the imaging features model to establish an integrated logistic model, and its diagnostic performance was assessed through ROC curve analysis. Statistical significance was set at *P* <0.05.

## 3 RESULTS

This study included 61 patients with IAC, classified into two groups: a moderately to well-differentiated group (n=40) and a poorly differentiated group (n=21). The cohort consisted of 30 males and 31 females, with ages ranging from 39 to 84 years (mean, 61±10 years). The average BMI was 24.71±3.36, the mean hematocrit was 39.33±5.20%, and the mean maximum lesion diameter was 1.88±0.67 cm.

No significant differences were observed between the two groups regarding nodule location, size, type, abnormal bronchus sign, bronchus-encapsulated air sign, or vascular abnormality (P > 0.05). However, significant differences were noted in maximum lesion diameter, presence of lobulation, spiculation, and pleural traction sign between the groups (*P* <0.05) (Table **1**).

Inter-observer agreement for qualitative imaging features (lobulation, spiculation, pleural traction sign, bronchus encapsulated air sign) was assessed using Cohen’s Kappa (0.76–0.82, substantial agreement), and for quantitative DECT parameters (ECV_A_, ECV_V_, IC_A_, IC_V_, NIC_A_, NIC_V_, nZeff_A_, nZeff_V_) using ICC (0.76–0.85, substantial to almost perfect agreement) based on a two-way random-effects model. These results confirm measurement reliability.

The moderately/well-differentiated group demonstrated significantly higher mean values of ECV_A_, NIC_A_, ECV_V_, IC_V_, NIC_V_, and nZeff_V_ compared to the poorly differentiated group (P<0.05) (Table **2**). No significant differences were observed between the groups for any other parameters (P > 0.05).

**Fig. (1):**
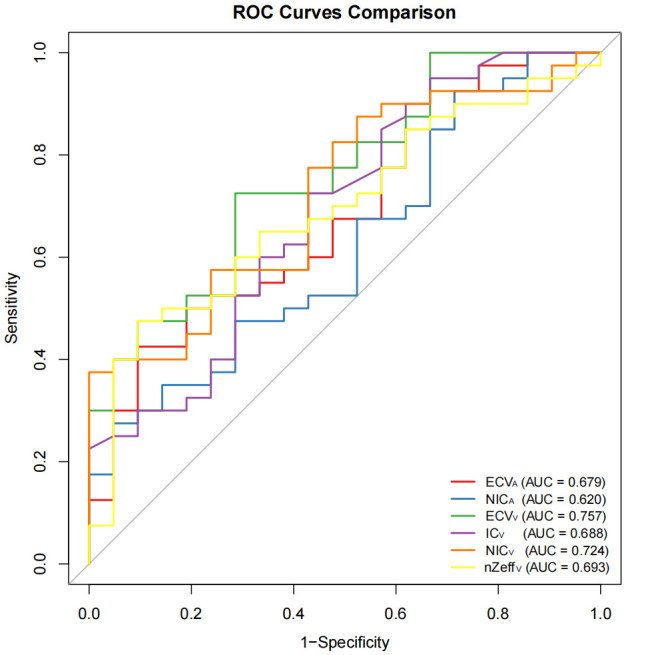
ROC curves for differentiating moderately to well-differentiated from poorly differentiated lung IAC: ECV_A_ (AUC = 0.679, 95% CI: 0.539–0.818), NIC_A_ (AUC = 0.620, 95% CI: 0.472–0.768), ECV_V_ (AUC = 0.757, 95% CI: 0.633–0.881), IC_V_ (AUC = 0.688, 95% CI: 0.543–0.833), NIC_V_ (AUC = 0.724, 95% CI: 0.593–0.855), nZ_effV_ (AUC = 0.693, 95% CI: 0.557–0.829).

ROC curve analysis was performed to evaluate the diagnostic performance of significant parameters in differentiating between moderately to well-differentiated and poorly differentiated IAC. The Areas Under the Curve (AUC) for ECV_A_, NIC_A_, ECV_V_, IC_V_, NIC_V_, and nZeff_V_ were 0.679, 0.620, 0.757, 0.688, 0.724 and 0.693, respectively (Fig. **1**). ECV_V_ demonstrated the highest diagnostic performance with an AUC of 0.757 (Table **3**), yielding a sensitivity of 72.5% and specificity of 71.4%.

**Fig. (2):**
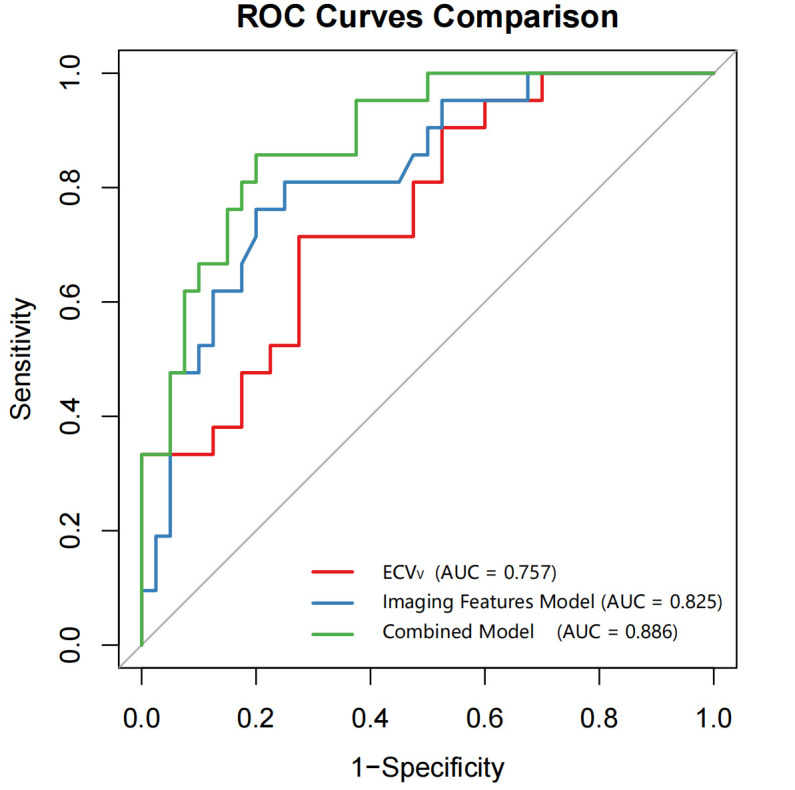
ROC curves comparing the diagnostic performance of three models for differentiating moderately to well-differentiated from poorly differentiated lung IAC: the ECV_V_ model (AUC = 0.757, 95% CI: 0.633–0.881), the imaging features model (AUC = 0.825, 95% CI: 0.717–0.933), and the combined model integrating ECV_V_ with imaging features (AUC = 0.886, 95% CI: 0.803–0.968).

Performance of Combined Model Using Conventional Imaging Features and ECV_V_ Five imaging features were selected as independent variables for the imaging features model using binary logistic regression: maximum lesion diameter, bronchus encapsulated air sign, lobulation, spiculation, and pleural traction sign, with multivariate Odds Ratios (ORs) of 2.00, 2.58, 1.88, 6.40, and 5.29, respectively. The resulting imaging model yielded an AUC of 0.825, with a sensitivity of 76.2% and specificity of 80.0% (Table **4**). A combined model incorporating both the imaging features (including spiculation and pleural traction sign) and ECV_V_ achieved an AUC of 0.886, with a sensitivity of 85.7% and specificity of 80.0% (Table **3** and Fig. **2**).

The formula is expressed as: Logit(P)=-0.769+0.692*Max_Diameter-0.170*ECV_V_+0.950*Bronchus_Encapsulated_Air_Sign+0.633*Lobulation+1.857*Spiculation+1.666*Pleural_Indentation

The combined model outperformed both the imaging-only model and the ECV_V_ model in terms of AUC, sensitivity, and specificity. These findings suggest that ECV_V_ provides complementary information to conventional imaging features, thereby significantly enhancing the diagnostic performance of the integrated model. Representative cases illustrating these findings are shown in Figs. (**3** and **4**).

## 4 DISCUSSION

Compared to conventional single-energy CT, DLCT offers significant advantages, particularly in its ability to provide multiple quantitative parameters for comprehensive lesion assessment. The commonly used DLCT parameters include IC, NIC, and Zeff. IC represents the mass or concentration of iodine elements per unit volume, enabling precise evaluation of lesion vascularity. Tumors typically exhibit higher iodine concentrations due to the process of neoangiogenesis. NIC, calculated as the ratio of lesion IC to aortic IC at the same level, helps normalize variations in contrast agent flow rates. Our results demonstrate that this normalization significantly enhances the differentiation between moderately to well-differentiated and poorly differentiated groups.

ECV, derived from NIC and hematocrit, quantitatively reflects features of the tumor microenvironment, especially those related to the extracellular matrix (ECM) [16]. ECV serves as a quantitative indicator of the proportion of extracellular space in tissues, exhibiting an inverse correlation with tumor cell density [17]. Well-differentiated lung adenocarcinomas have distinct pathological characteristics, including organized glandular structures with a regular cell arrangement and a lower cell density [18], While poorly differentiated adenocarcinomas exhibit loss of glandular architecture, characterized by disorganized and dense cellular patterns [19].

Our study revealed that venous phase ECV effectively differentiates between moderately/well-differentiated and poorly differentiated IAC, with poorly differentiated IAC showing significantly lower ECV values. This finding can be attributed to the dense cellular arrangement and reduced extracellular space in poorly differentiated IAC, coupled with their primary blood supply from the bronchial arterial system, resulting in relatively poor perfusion compared to normal lung tissue [20]. The lack of statistical significance in some arterial phase DLCT parameters may be attributed to insufficient contrast agent distribution within the lesion's extracellular space, resulting from shorter delay times [21]. Research suggests that venous phase hemodynamics are more stable than arterial phase, potentially providing more accurate tumor characterization [22].

The results showed that spiculation and pleural traction signs were independent predictors for differentiating between groups. A simplified model combining ECV_V_, spiculation, and pleural traction sign was further evaluated to explore the diagnostic potential of a minimal set of features. As typical morphological characteristics of poorly differentiated IAC, spiculation and pleural traction sign, when integrated with the quantitative parameter ECV_V_, demonstrated high diagnostic efficiency and may be applicable in clinical settings that require rapid assessment.

The venous phase effective atomic number derived from DLCT showed significant diagnostic value. In dual-energy CT, the “effective atomic number” is calculated from tissue absorption measurements at low and high X-ray energies, representing an equivalent atomic number that reflects a material's X-ray absorption characteristics [23]. Our study utilized nZ_eff_, defined as the ratio of lesion Z_eff_ to aortic Z_eff_, to minimize the effects of contrast flow rate [24]. The significant difference in nZeff between groups indicates its potential value in pathological grading of lung adenocarcinoma.

## 5 LIMITATIONS

Several limitations should be acknowledged. As a retrospective study conducted at a single center with a limited sample size, the statistical power may be insufficient. Future multicenter studies with larger cohorts are warranted to validate the robustness of our findings. Additionally, the study specifically addressed the clinical challenge of pathological grading of pulmonary nodules. As a result, only part-solid or solid invasive adenocarcinomas measuring 1–3 cm in diameter were included, which may limit the generalizability of the results.

## CONCLUSION

Our findings suggest that ECV, combined with conventional imaging features, is a promising non-invasive tool for differentiating pathological grades of lung invasive adenocarcinoma, achieving an AUC of 0.886. This integrated model enhances diagnostic accuracy, potentially reducing reliance on invasive biopsies. Validation in larger, multicenter cohorts is needed to confirm its clinical utility.

## Figures and Tables

**Fig. (3):**
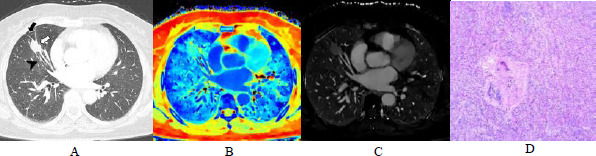
A 60-year-old female with a solid nodule in the middle lobe of the right lung, pathologically diagnosed as poorly differentiated adenocarcinoma. **A**). The lung window image shows pleural retraction (black arrow), the lobulation sign (white arrow), and an abnormal vascular sign (arrowhead). **B**). Venous phase effective atomic number map demonstrating an average nZeff_V_ of 0.87. **C**). Venous phase iodine density map showing average IC_V_ of 2.33 mg/ml, NIC_V_ of 0.06, and ECV_V_ of 16.35%, which is below the diagnostic threshold of 21.05%. **D**). Histopathological image (H&E, ×100) revealing extensive high-grade invasive components (solid and micropapillary patterns).

**Fig. (4):**
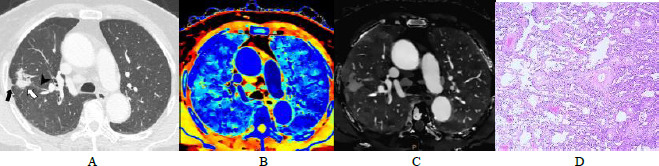
A 78-year-old female with a solid nodule in the upper lobe of the right lung, pathologically diagnosed as moderately differentiated adenocarcinoma. **A**). Lung window image shows pleural retraction (black arrow), lobulation sign (white arrow), and abnormal bronchus sign (arrowhead). **B**). Venous phase effective atomic number map demonstrating an average nZeff_V_ of 0.92. **C**). Venous phase iodine density map showing average IC_V_ of 1.41 mg/ml, NIC_V_ of 0.27, and ECV_V_ of 21.97%, which is above the diagnostic threshold of 21.05%. **D**). Histopathological image (H&E, ×100) showing predominantly acinar pattern.

**Table 1:** Comparison of clinical and conventional imaging features between moderately/well-differentiated and poorly differentiated groups.

**Characteristics**	**Moderately/Well-differentiated Group (n=40)**	**Poorly Differentiated Group (n=21)**	**P**
Age (years)	62.20±10.37	59.90±10.62	0.419
Sex (Male/Female)	17/23	13/8	0.150
BMI	24.95±3.30	24.26±3.52	0.447
Hematocrit (%)	39.41±5.16	39.19±5.39	0.873
Nodule Location	-	-	0.438
Right Upper Lobe	12(30.00)	5(23.81)	-
Right Middle Lobe	3(7.50)	2(9.52)	-
Right Lower Lobe	4(10.00)	6(28.57)	-
Left Upper Lobe	18(45.00)	7(33.33)	-
Left Lower Lobe	3(7.50)	1(4.76)	-
Maximum Lesion Diameter (cm)	1.75±0.69	2.13±0.57	0.036
Subsolid Nodule (%)	17(42.50)	5(23.81)	0.149
Lobulation(%)	16(40.00)	14(66.67)	0.048
Spiculation(%)	14(35.00)	14(66.67)	0.018
Abnormal Bronchus Sign(%)	21(52.50)	14(66.67)	0.288
Pleural Traction Sign (%)	15(37.50)	14(66.67)	0.030
Bronchus encapsulated air sign(%)	4(10.00)	6(28.57)	0.063
Vascular Convergence(%)	27(67.50)	16(76.19)	0.480

**Note:** Data are mean ± standard deviation or n (%). *P* values from t-test (continuous) and chi-square test (categorical). *P* values < 0.05 were considered statistically significant.

**Table 2:** Comparison of ECV and other DECT parameters between moderately/well-differentiated and poorly differentiated IAC groups.

**Group**	**IC_A_(mg/ml)**	**ECV_A_(%)**	**NIC_A_**	**nZ_effA_**	**IC_V_(mg/ml)**	**ECV_V_(%)**	**NIC_V_**	**nZ_effV_**
Moderately/Well-differentiated Group (n=40)	1.65±0.63	7.59±3.77	0.14±0.07	0.72±0.06	2.10±0.49	24.77±7.61	0.45±0.12	0.91±0.04
Poorly Differentiated Group (n=21)	1.45±0.72	5.26±2.73	0.10±0.05	0.70±0.05	1.76±0.46	17.95±5.36	0.36±0.09	0.88±0.03
*P*	0.270	0.015	0.034	0.184	0.011	0.001	0.002	0.023

**Note:** Data are presented as mean ± standard deviation. *P* values from t-test (continuous) *P* values < 0.05 were considered statistically significant.

**Table 3:** Diagnostic performance of IC_V_, ECV_V_, nZeff_V_, and NIC_V_ for poorly differentiated invasive adenocarcinoma: AUC, Cut-off, sensitivity, specificity, and youden index.

**Parameter**	**AUC(95% CI)**	**Cut-off**	**Sensitivity**	**Specificity**	**Youden Index**
ECV_A_	0.679(0.539-0.818)	6.925	0.425	0.905	0.330
NIC_A_	0.620(0.472-0.768)	0.172	0.275	0.952	0.227
ECV_V_	0.757(0.633-0.881)	21.050	0.725	0.714	0.439
IC_V_	0.688(0.543-0.833)	1.673	0.825	0.476	0.301
NIC_V_	0.724(0.593-0.855)	0.497	0.375	1.000	0.375
nZ_effV_	0.693(0.557-0.829)	0.918	0.475	0.905	0.380
Imaging Features Model	0.825(0.717-0.933)	0.398	0.762	0.800	0.562
Combined Model	0.886(0.803-0.968)	0.331	0.857	0.800	0.657

**Note:** Data are presented as the area under the receiver operating characteristic curve, along with 95% Confidence Intervals (CIs), cut-off values, sensitivities, specificities, and Youden indices for each parameter. Abbreviations: ECV_A_, arterial phase extracellular volume fraction; NIC_A_, arterial phase normalized iodine concentration; ECV_V_, venous phase extracellular volume fraction; IC_V_, iodine concentration in venous phase; NIC_V_, normalized iodine concentration in venous phase; nZeff_V_, normalized effective atomic number in venous phase.

**Table 4:** Univariate and multivariate logistic regression analysis of features distinguishing poorly differentiated from moderately/well-differentiated adenocarcinoma.

**Variables**	**OR (Univariate)**	**P**	**OR (Multivariate)**	**P**
Maximum Lesion Diameter	2.37(1.04-5.40)	0.040	2.00(0.64-6.28)	0.236
Venous Phase ECV	0.85(0.76-0.94)	0.002	0.84(0.74-0.96)	0.010
Bronchus Encapsulated Air Sign	3.60(0.89-14.62)	0.073	2.58(0.40-16.85)	0.321
Abnormal Bronchus Sign	1.81(0.60-5.43)	0.290	-	-
Vascular Convergence	1.54(0.46-5.13)	0.481	-	-
Lobulation	3.00(0.99-9.07)	0.052	1.88(0.43-8.17)	0.398
Spiculation	3.71(1.22-11.34)	0.021	6.40(1.29-31.80)	0.023
Pleural Traction Sign	3.33(1.10-10.12)	0.034	5.29(1.05-26.59)	0.043

**Note:** Data are presented as Odds Ratios (OR) with 95% confidence intervals (in parentheses). *P* values < 0.05 were considered statistically significant. Variables with *P* < 0.10 in univariate analysis were included in the multivariate model. Abbreviations: ECV, extracellular volume fraction.

## Data Availability

The data and supportive information is available within the article.

## LIST OF ABBREVIATIONS

AUC: Area Under the Curve
BMI: Body Mass Index
CI: Confidence Interval
CT: Computed Tomography
DECT: Dual-Energy Computed Tomography
DLCT: Dual-Layer Detector Spectral CT
ECV: Extracellular Volume Fraction
ECV_A_: Extracellular Volume Fraction in Arterial phase
ECV_V_: Extracellular Volume Fraction in Venous phase
IAC: Invasive Adenocarcinoma
IC: Iodine Concentration
IC_A_: Iodine Concentration Value in Arterial phase
IC_V_: Iodine Concentration Value in Venous phase
NIC_A_: Normalized Iodine Concentration in Arterial phase
NIC_V_: Normalized Iodine Concentration in Venous phase
nZeff_A_: Normalized Effective Atomic Number in Arterial phase
nZeff_V_: Normalized Effective Atomic Number in Venous phase
OR: Odds Ratio
ROC: Receiver Operating Characteristic
Zeff: Effective Atomic Number

## STANDARDS OF REPORTING

STROBE guidelines were followed.
